# Response to letter regarding maternal and fetal outcomes across hypertensive pregnancy subtypes

**DOI:** 10.1016/j.ijcrp.2025.200506

**Published:** 2025-09-01

**Authors:** Laith Alhuneafat, Fares Ghanem, Sneha Nandy, Sana Khan, Anushree Puttur, Ahmad Jabri, Alaq Haddad, Bhavadharini Ramu, Bethany Sabol, Jessica Schultz, Selma Carlson

**Affiliations:** a Division of Cardiovascular Disease, University of Minnesota, Minneapolis, MN, USA; b Department of Cardiovascular Medicine, Southern Illinois University, Springfield, IL, USA; c Department of Medicine, Allegheny Health Network, Pittsburgh, PA, USA; d Department of Cardiovascular Disease, Henry Ford Health System, Detroit, MI, USA; e School of Public Health, Harvard University, Boston, MA, USA; f Department of Maternal-Fetal Medicine, University of Minnesota, Minneapolis, MN, USA

We thank Drs. Ma and Tang for their thoughtful commentary on our article, “Examining maternal and fetal outcomes across various subtypes of hypertension during pregnancy.” [1] Their letter raises four issues: residual confounding, diagnostic misclassification, multiplicity of testing, and causal inference. Many of these limitations were acknowledged in our manuscript, however merits clarification.

First, residual confounding is an inherent limitation of retrospective studies using administrative data like the NIS. While such databases cannot capture every behavioral or biometric covariate, we minimized confounding with a multivariable model incorporating comorbidities, demographics, and hospital characteristics.

Second, regarding diagnostic codes, we restricted our study to 2016–2020 to avoid ICD-9 transitions and used established ICD-10-CM codes aligned with national guidelines. A recent validation study showed these codes have high predictive values and specificity (>90 %) and moderate to high sensitivity (>80 %) for most hypertensive subtypes [2]. Our study period also coincided with ACOG updates broadening preeclampsia criteria, which may increase coding of cases without proteinuria [3]. These changes likely affected coding practices and increased detection of subtypes like preeclampsia without proteinuria, reflecting real-world shifts in documentation and diagnosis.

Third, while multiple comparisons may raise type I error concerns, our hypothesis-driven analyses targeted distinct outcomes across clinically meaningful subtypes and were contextualized with findings from prior studies [4,5].

Finally, we agree causal inference is inappropriate with NIS data. We used association-based language and highlighted this in our limitations. We believe our conclusions remain valid within these discussed parameters. We appreciate their comments and insights for future research.

## CRediT authorship contribution statement

**Laith Alhuneafat:** Writing – original draft. **Fares Ghanem:** Writing – review & editing. **Sneha Nandy:** Writing – review & editing. **Sana Khan:** Writing – review & editing. **Anushree Puttur:** Writing – review & editing. **Ahmad Jabri:** Writing – review & editing. **Alaq Haddad:** Writing – review & editing. **Bhavadharini Ramu:** Writing – review & editing. **Bethany Sabol:** Writing – review & editing. **Jessica Schultz:** Writing – review & editing. **Selma Carlson:** Validation, Writing – review & editing.

## Data availability

The National Inpatient Sample (NIS) is a large publicly available all-payer database in the United States. Healthcare Cost and Utilization Project Agency for Healthcare Research and Quality (HCUP AHRQ) has restrictions on data sharing based on a Data Use Agreement any questions should be directed to them.

## Ethical compliance

The research qualifies as no risk or minimal risk to subjects and our institution does not require ethical approval for NIS database studies.

## Study funding

No funding applied.

## Declaration of competing interest

The authors declare the following financial interests/personal relationships that may be considered as potential competing interests:BR: Speaker's honoraria: Abbott Inc.All other authors have nothing to disclose.
